# Methyl 2-[4-(4-chloro­benzo­yl)phen­oxy]-2-methyl­propano­ate

**DOI:** 10.1107/S1600536812019812

**Published:** 2012-05-12

**Authors:** Baohua Zou, Zheng Fang, Hui Zhong, Kai Guo, Ping Wei

**Affiliations:** a School of Pharmaceutical Sciences, Nanjing University of Technology, Puzhunan Road No. 30 Nanjing, Nanjing 210009, People’s Republic of China; b College of Life Science and Pharmaceutical Engineering, Nanjing University of Technology, Puzhunan Road No. 5 Nanjing, Nanjing 210009, People’s Republic of China

## Abstract

In the title compound, C_18_H_17_ClO_4_, the dihedral angle between the mean planes of the benzene rings is 53.4 (1)°. Weak inter­molecular C—H⋯O inter­actions are observed.

## Related literature

For background, see: Guichard *et al.* (2000 ▶). For the synthesis of the title compound, see: Bandgar *et al.* (2011 ▶). For bond lengths, see: Allen *et al.* (1987 ▶).
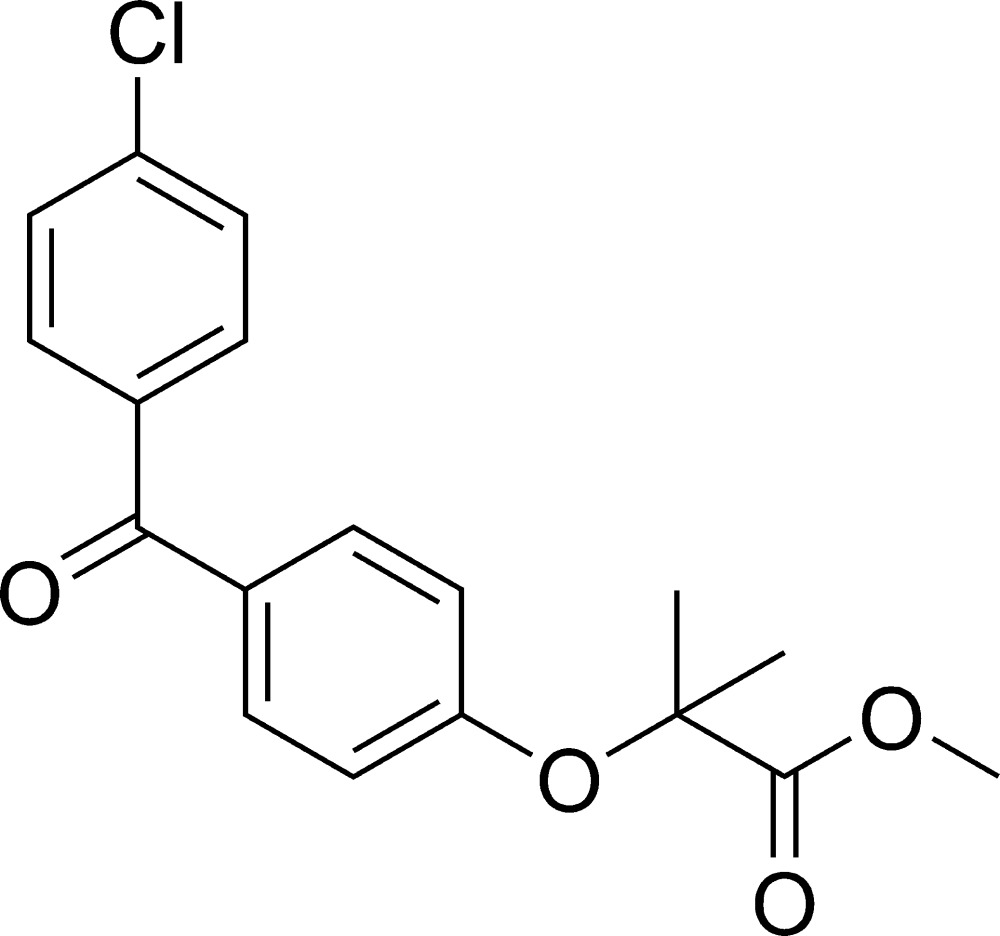


## Experimental

### 

#### Crystal data

C_18_H_17_ClO_4_
*M* *_r_* = 332.77
Orthorhombic, 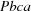

*a* = 19.657 (4) Å
*b* = 7.5860 (15) Å
*c* = 22.436 (5) Å
*V* = 3345.6 (12) Å^3^
*Z* = 8
Mo *K*α radiation
μ = 0.25 mm^−1^
*T* = 293 K
0.30 × 0.20 × 0.10 mm

#### Data collection

Enraf–Nonius CAD-4 diffractometer
Absorption correction: ψ scan (North *et al.*, 1968 ▶) *T*_min_ = 0.930, *T*_max_ = 0.976
6018 measured reflections
3065 independent reflections
1432 reflections with *I* > 2σ(*I*)
*R*_int_ = 0.092

#### Refinement

*R*[*F* ^2^ > 2σ(*F* ^2^)] = 0.067
*wR*(*F* ^2^) = 0.194
*S* = 1.00
3065 reflections
208 parameters
H-atom parameters constrained
Δρ_max_ = 0.20 e Å^−3^
Δρ_min_ = −0.21 e Å^−3^

### 

Data collection: *CAD-4 EXPRESS* (Enraf–Nonius, 1989 ▶); cell refinement: *CAD-4 EXPRESS*; data reduction: *XCAD4* (Harms & Wocadlo, 1995 ▶); program(s) used to solve structure: *SHELXS97* (Sheldrick, 2008 ▶); program(s) used to refine structure: *SHELXL97* (Sheldrick, 2008 ▶); molecular graphics: *SHELXTL-Plus* (Sheldrick, 2008 ▶); software used to prepare material for publication: *SHELXL97*.

## Supplementary Material

Crystal structure: contains datablock(s) global, I. DOI: 10.1107/S1600536812019812/jj2129sup1.cif

Structure factors: contains datablock(s) I. DOI: 10.1107/S1600536812019812/jj2129Isup2.hkl

Supplementary material file. DOI: 10.1107/S1600536812019812/jj2129Isup3.cml

Additional supplementary materials:  crystallographic information; 3D view; checkCIF report

## Figures and Tables

**Table 1:** Hydrogen-bond geometry (Å, °)

*D*—H⋯*A*	*D*—H	H⋯*A*	*D*⋯*A*	*D*—H⋯*A*
C1—H1*A*⋯O3^i^	0.93	2.37	3.254 (6)	159

Symmetry code: (i) 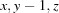.

**Fig. 1.**
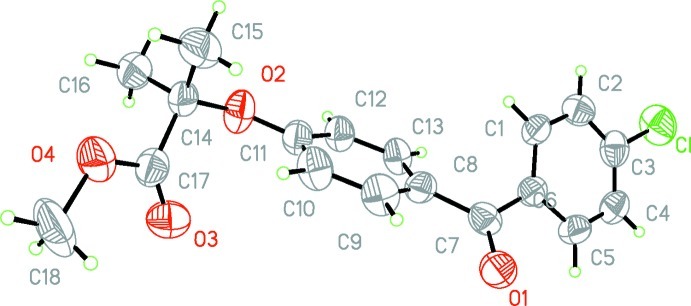
The molecular structure of the title compound, (I), showing the atom labeling scheme and 50% probability displacement ellipsoids.

**Fig. 2.**
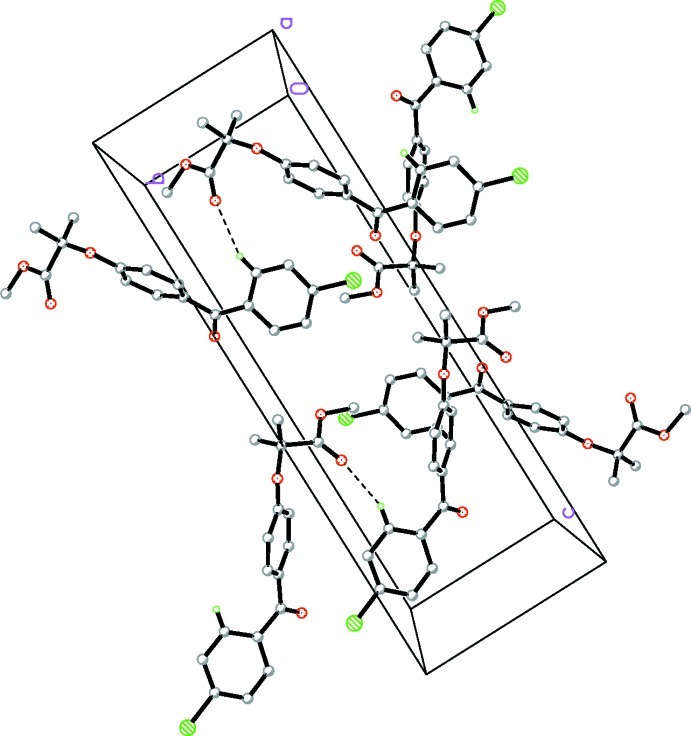
Packing diagram of the title compound viewed along the a axis. Dashed lines indicate weak C—H···O intermolecular interactions. Remaining H atoms have been omitted for clarity.

## supplementary crystallographic information

### Comment

The title compound, C_18_H_17_ClO_4_, (I), is a derivative of Fenofibrate, a antihypertensive drug (Guichard *et al.* 2000). We report herein its crystal structure.

In the title compound, (I), the dihedral angle between the mean planes of the benzene and phenyl rings is 53.4 (1)°. Crystal packing is influenced by weak C—H···O intermolecular interactions. Bond lengths are in normal ranges (Allen *et al.* 1987).

### Experimental

2-(4-(4-chlorobenzoyl)phenoxy)-2-methylpropanoic acid (6.28 mmol, 2.00 g) was dissolved in 35% hydrochloric acid methanol solution (15 mL), the solution was heated to 338.15 K under N2 atmosphere for 3 h. The reaction mexture was cooled to room temperature and the solvent was distilled to get the crude compound. The crude compond was dissolved in dichloromethane (15 mL), washed with water (10 mL) three times, dried, and concentrated to get the title compound (1.95 g). pure: white solid (Bandgar *et al.* 2011). Crystals of the title compound suitable for X-ray diffraction were obtained by slow evaporation of an ethanol solution.

### Refinement

H atoms were positioned geometrically with C—H = 0.93, 0.98 and 0.97 Å for aromatic, methine and methylene H atoms, respectively, and constrained to ride on their parent atoms, with *U*_iso_(H) = 1.2 (or 1.5 for methyl groups) times *U*_eq_(C).

### Crystal data

**Table:**

C_18_H_17_ClO_4_	*F*(000) = 1392
*M**_r_* = 332.77	*D*_x_ = 1.321 Mg m^−^^3^
Orthorhombic, *P**b**c**a*	Mo *K*α radiation, λ = 0.71073 Å
Hall symbol: -P 2ac 2ab	Cell parameters from 25 reflections
*a* = 19.657 (4) Å	θ = 9–12°
*b* = 7.5860 (15) Å	µ = 0.25 mm^−^^1^
*c* = 22.436 (5) Å	*T* = 293 K
*V* = 3345.6 (12) Å^3^	Block, colorless
*Z* = 8	0.30 × 0.20 × 0.10 mm

### Data collection

**Table:**

Enraf–Nonius CAD-4 diffractometer	3065 independent reflections
Radiation source: fine-focus sealed tube	1432 reflections with *I* > 2σ(*I*)
Graphite monochromator	*R*_int_ = 0.092
Detector resolution: 16.0355 pixels mm^-1^	θ_max_ = 25.4°, θ_min_ = 1.8°
ω/2θ scans	*h* = 0→23
Absorption correction: ψ scan (North *et al.*, 1968)	*k* = 0→9
*T*_min_ = 0.930, *T*_max_ = 0.976	*l* = −27→27
6018 measured reflections	

### Refinement

**Table:**

Refinement on *F*^2^	Primary atom site location: structure-invariant direct methods
Least-squares matrix: full	Secondary atom site location: difference Fourier map
*R*[*F*^2^ > 2σ(*F*^2^)] = 0.067	Hydrogen site location: inferred from neighbouring sites
*wR*(*F*^2^) = 0.194	H-atom parameters constrained
*S* = 1.00	*w* = 1/[σ^2^(*F*_o_^2^) + (0.070*P*)^2^ + 1.*P*] where *P* = (*F*_o_^2^ + 2*F*_c_^2^)/3
3065 reflections	(Δ/σ)_max_ < 0.001
208 parameters	Δρ_max_ = 0.20 e Å^−^^3^
0 restraints	Δρ_min_ = −0.21 e Å^−^^3^

### Special details

**Table:**

Geometry. All e.s.d.'s (except the e.s.d. in the dihedral angle between two l.s. planes) are estimated using the full covariance matrix. The cell e.s.d.'s are taken into account individually in the estimation of e.s.d.'s in distances, angles and torsion angles; correlations between e.s.d.'s in cell parameters are only used when they are defined by crystal symmetry. An approximate (isotropic) treatment of cell e.s.d.'s is used for estimating e.s.d.'s involving l.s. planes.
Refinement. Refinement of *F*^2^ against ALL reflections. The weighted *R*-factor *wR* and goodness of fit *S* are based on *F*^2^, conventional *R*-factors *R* are based on *F*, with *F* set to zero for negative *F*^2^. The threshold expression of *F*^2^ > σ(*F*^2^) is used only for calculating *R*-factors(gt) *etc*. and is not relevant to the choice of reflections for refinement. *R*-factors based on *F*^2^ are statistically about twice as large as those based on *F*, and *R*- factors based on ALL data will be even larger.

### Fractional atomic coordinates and isotropic or equivalent isotropic displacement parameters (Å^2^)

**Table:**

	*x*	*y*	*z*	*U*_iso_*/*U*_eq_	
Cl	0.19155 (8)	0.28985 (19)	0.10094 (7)	0.0861 (5)	
O1	0.03598 (16)	1.0570 (4)	0.15179 (15)	0.0650 (10)	
O2	0.18493 (16)	1.3480 (4)	0.38182 (14)	0.0641 (9)	
O3	0.1333 (2)	1.6697 (5)	0.34807 (16)	0.0758 (11)	
O4	0.07842 (17)	1.7018 (4)	0.43384 (15)	0.0688 (10)	
C1	0.1314 (2)	0.6897 (6)	0.2032 (2)	0.0513 (12)	
H1A	0.1311	0.7155	0.2437	0.062*	
C2	0.1552 (2)	0.5266 (6)	0.1842 (2)	0.0569 (12)	
H2A	0.1694	0.4429	0.2119	0.068*	
C3	0.1576 (2)	0.4899 (7)	0.1245 (2)	0.0576 (13)	
C4	0.1335 (3)	0.6088 (7)	0.0831 (2)	0.0627 (13)	
H4A	0.1341	0.5815	0.0427	0.075*	
C5	0.1087 (2)	0.7686 (7)	0.1024 (2)	0.0579 (13)	
H5A	0.0917	0.8481	0.0745	0.069*	
C6	0.1083 (2)	0.8146 (6)	0.16303 (18)	0.0451 (11)	
C7	0.0807 (2)	0.9881 (6)	0.1814 (2)	0.0496 (11)	
C8	0.1063 (2)	1.0766 (6)	0.23639 (19)	0.0461 (11)	
C9	0.0652 (2)	1.1914 (6)	0.2664 (2)	0.0583 (13)	
H9A	0.0207	1.2071	0.2534	0.070*	
C10	0.0875 (2)	1.2857 (6)	0.3158 (2)	0.0576 (12)	
H10A	0.0584	1.3619	0.3359	0.069*	
C11	0.1539 (2)	1.2636 (6)	0.33445 (19)	0.0502 (11)	
C12	0.1967 (2)	1.1497 (6)	0.3040 (2)	0.0523 (12)	
H12A	0.2417	1.1368	0.3162	0.063*	
C13	0.1730 (2)	1.0555 (6)	0.2559 (2)	0.0519 (11)	
H13A	0.2017	0.9773	0.2363	0.062*	
C14	0.1484 (2)	1.4503 (6)	0.42572 (19)	0.0488 (11)	
C15	0.0947 (3)	1.3430 (6)	0.4578 (2)	0.0746 (16)	
H15A	0.1154	1.2408	0.4753	0.112*	
H15B	0.0604	1.3066	0.4299	0.112*	
H15C	0.0742	1.4134	0.4885	0.112*	
C16	0.2051 (2)	1.5112 (7)	0.4665 (2)	0.0657 (14)	
H16A	0.2249	1.4110	0.4861	0.099*	
H16B	0.1870	1.5904	0.4959	0.099*	
H16D	0.2392	1.5707	0.4435	0.099*	
C17	0.1192 (2)	1.6172 (6)	0.3962 (2)	0.0544 (12)	
C18	0.0490 (3)	1.8640 (6)	0.4124 (3)	0.096 (2)	
H18D	0.0206	1.9141	0.4428	0.143*	
H18A	0.0223	1.8405	0.3775	0.143*	
H18B	0.0847	1.9454	0.4026	0.143*	

### Atomic displacement parameters (Å^2^)

**Table:**

	*U*^11^	*U*^22^	*U*^33^	*U*^12^	*U*^13^	*U*^23^
Cl	0.1045 (12)	0.0681 (9)	0.0857 (11)	0.0090 (8)	0.0163 (9)	−0.0196 (8)
O1	0.055 (2)	0.055 (2)	0.085 (2)	−0.0014 (17)	−0.0228 (18)	0.0061 (18)
O2	0.058 (2)	0.069 (2)	0.065 (2)	0.0090 (17)	−0.0062 (16)	−0.0251 (18)
O3	0.095 (3)	0.070 (2)	0.063 (2)	−0.005 (2)	0.008 (2)	0.0145 (19)
O4	0.081 (2)	0.054 (2)	0.072 (2)	0.0153 (18)	0.008 (2)	−0.0068 (18)
C1	0.058 (3)	0.057 (3)	0.039 (2)	−0.009 (2)	−0.005 (2)	−0.001 (2)
C2	0.059 (3)	0.051 (3)	0.060 (3)	0.004 (2)	0.000 (2)	0.003 (2)
C3	0.055 (3)	0.060 (3)	0.058 (3)	−0.006 (2)	0.008 (2)	−0.014 (3)
C4	0.080 (4)	0.065 (3)	0.043 (3)	−0.009 (3)	−0.005 (3)	−0.009 (3)
C5	0.067 (3)	0.064 (3)	0.043 (3)	−0.010 (3)	−0.007 (2)	0.010 (2)
C6	0.043 (2)	0.051 (3)	0.041 (2)	−0.008 (2)	−0.001 (2)	0.003 (2)
C7	0.045 (2)	0.042 (3)	0.061 (3)	0.002 (2)	−0.001 (2)	0.004 (2)
C8	0.041 (2)	0.049 (3)	0.048 (3)	−0.003 (2)	−0.006 (2)	0.003 (2)
C9	0.041 (2)	0.059 (3)	0.075 (3)	0.004 (2)	−0.001 (2)	−0.005 (3)
C10	0.043 (2)	0.053 (3)	0.076 (3)	0.007 (2)	0.006 (3)	−0.012 (3)
C11	0.052 (3)	0.048 (3)	0.051 (3)	−0.001 (2)	−0.005 (2)	−0.006 (2)
C12	0.046 (2)	0.049 (3)	0.062 (3)	0.004 (2)	−0.006 (2)	−0.009 (2)
C13	0.044 (3)	0.044 (3)	0.067 (3)	0.001 (2)	−0.002 (2)	−0.011 (2)
C14	0.055 (3)	0.045 (3)	0.046 (3)	−0.006 (2)	−0.003 (2)	−0.006 (2)
C15	0.095 (4)	0.057 (3)	0.072 (4)	−0.009 (3)	0.013 (3)	0.007 (3)
C16	0.078 (3)	0.060 (3)	0.059 (3)	0.001 (3)	−0.013 (3)	−0.004 (3)
C17	0.059 (3)	0.055 (3)	0.049 (3)	−0.005 (2)	−0.002 (2)	−0.008 (3)
C18	0.105 (5)	0.048 (3)	0.134 (6)	0.028 (3)	−0.013 (4)	−0.021 (4)

### Geometric parameters (Å, º)

**Table:**

Cl—C3	1.740 (5)	C9—C10	1.390 (6)
O1—C7	1.219 (5)	C9—H9A	0.9300
O2—C11	1.382 (5)	C10—C11	1.381 (6)
O2—C14	1.445 (5)	C10—H10A	0.9300
O3—C17	1.183 (5)	C11—C12	1.386 (6)
O4—C17	1.330 (5)	C12—C13	1.377 (6)
O4—C18	1.442 (6)	C12—H12A	0.9300
C1—C6	1.385 (6)	C13—H13A	0.9300
C1—C2	1.390 (6)	C14—C15	1.514 (6)
C1—H1A	0.9300	C14—C16	1.515 (6)
C2—C3	1.370 (6)	C14—C17	1.539 (6)
C2—H2A	0.9300	C15—H15A	0.9600
C3—C4	1.378 (7)	C15—H15B	0.9600
C4—C5	1.376 (6)	C15—H15C	0.9600
C4—H4A	0.9300	C16—H16A	0.9600
C5—C6	1.405 (6)	C16—H16B	0.9600
C5—H5A	0.9300	C16—H16D	0.9600
C6—C7	1.482 (6)	C18—H18D	0.9600
C7—C8	1.493 (6)	C18—H18A	0.9600
C8—C9	1.367 (6)	C18—H18B	0.9600
C8—C13	1.391 (5)		
			
C11—O2—C14	123.6 (3)	O2—C11—C12	113.5 (4)
C17—O4—C18	116.2 (4)	C13—C12—C11	120.3 (4)
C6—C1—C2	121.3 (4)	C13—C12—H12A	119.9
C6—C1—H1A	119.3	C11—C12—H12A	119.9
C2—C1—H1A	119.3	C12—C13—C8	120.4 (4)
C3—C2—C1	119.5 (5)	C12—C13—H13A	119.8
C3—C2—H2A	120.3	C8—C13—H13A	119.8
C1—C2—H2A	120.3	O2—C14—C15	112.4 (4)
C2—C3—C4	121.0 (5)	O2—C14—C16	102.1 (3)
C2—C3—Cl	119.2 (4)	C15—C14—C16	112.9 (4)
C4—C3—Cl	119.9 (4)	O2—C14—C17	109.5 (4)
C5—C4—C3	119.2 (4)	C15—C14—C17	112.8 (4)
C5—C4—H4A	120.4	C16—C14—C17	106.4 (3)
C3—C4—H4A	120.4	C14—C15—H15A	109.5
C4—C5—C6	121.6 (4)	C14—C15—H15B	109.5
C4—C5—H5A	119.2	H15A—C15—H15B	109.5
C6—C5—H5A	119.2	C14—C15—H15C	109.5
C1—C6—C5	117.4 (4)	H15A—C15—H15C	109.5
C1—C6—C7	123.2 (4)	H15B—C15—H15C	109.5
C5—C6—C7	119.4 (4)	C14—C16—H16A	109.5
O1—C7—C6	119.6 (4)	C14—C16—H16B	109.5
O1—C7—C8	120.0 (4)	H16A—C16—H16B	109.5
C6—C7—C8	120.4 (4)	C14—C16—H16D	109.5
C9—C8—C13	118.4 (4)	H16A—C16—H16D	109.5
C9—C8—C7	119.6 (4)	H16B—C16—H16D	109.5
C13—C8—C7	121.8 (4)	O3—C17—O4	123.9 (5)
C8—C9—C10	122.3 (4)	O3—C17—C14	125.6 (5)
C8—C9—H9A	118.9	O4—C17—C14	110.4 (4)
C10—C9—H9A	118.9	O4—C18—H18D	109.5
C11—C10—C9	118.6 (4)	O4—C18—H18A	109.5
C11—C10—H10A	120.7	H18D—C18—H18A	109.5
C9—C10—H10A	120.7	O4—C18—H18B	109.5
C10—C11—O2	126.4 (4)	H18D—C18—H18B	109.5
C10—C11—C12	120.0 (4)	H18A—C18—H18B	109.5
			
C6—C1—C2—C3	1.8 (7)	C9—C10—C11—O2	179.0 (4)
C1—C2—C3—C4	−3.3 (7)	C9—C10—C11—C12	0.1 (7)
C1—C2—C3—Cl	176.7 (4)	C14—O2—C11—C10	11.3 (7)
C2—C3—C4—C5	1.9 (7)	C14—O2—C11—C12	−169.7 (4)
Cl—C3—C4—C5	−178.1 (4)	C10—C11—C12—C13	−1.2 (7)
C3—C4—C5—C6	1.1 (7)	O2—C11—C12—C13	179.7 (4)
C2—C1—C6—C5	1.1 (6)	C11—C12—C13—C8	1.5 (7)
C2—C1—C6—C7	178.2 (4)	C9—C8—C13—C12	−0.5 (7)
C4—C5—C6—C1	−2.5 (6)	C7—C8—C13—C12	174.8 (4)
C4—C5—C6—C7	−179.8 (4)	C11—O2—C14—C15	58.5 (5)
C1—C6—C7—O1	−147.6 (4)	C11—O2—C14—C16	179.8 (4)
C5—C6—C7—O1	29.5 (6)	C11—O2—C14—C17	−67.7 (5)
C1—C6—C7—C8	31.1 (6)	C18—O4—C17—O3	1.8 (7)
C5—C6—C7—C8	−151.8 (4)	C18—O4—C17—C14	178.3 (4)
O1—C7—C8—C9	25.5 (6)	O2—C14—C17—O3	−11.3 (6)
C6—C7—C8—C9	−153.2 (4)	C15—C14—C17—O3	−137.3 (5)
O1—C7—C8—C13	−149.8 (4)	C16—C14—C17—O3	98.3 (5)
C6—C7—C8—C13	31.5 (6)	O2—C14—C17—O4	172.2 (3)
C13—C8—C9—C10	−0.6 (7)	C15—C14—C17—O4	46.3 (5)
C7—C8—C9—C10	−176.1 (4)	C16—C14—C17—O4	−78.1 (5)
C8—C9—C10—C11	0.9 (7)		

### Hydrogen-bond geometry (Å, º)

**Table:**

*D*—H···*A*	*D*—H	H···*A*	*D*···*A*	*D*—H···*A*
C1—H1*A*···O3^i^	0.93	2.37	3.254 (6)	159

Symmetry code: (i) *x*, *y*−1, *z*.
